# Effects of Arbuscular Mycorrhizal Fungus on Sodium and Chloride Ion Channels of *Casuarina glauca* under Salt Stress

**DOI:** 10.3390/ijms24043680

**Published:** 2023-02-12

**Authors:** Yihan Wang, Fengxin Dong, Hui Chen, Tingying Xu, Ming Tang

**Affiliations:** 1 State Key Laboratory of Conservation and Utilization of Subtropical Agro-Bioresources, Guangdong Laboratory for Lingnan Modern Agriculture, College of Forestry and Landscape Architecture, South China Agricultural University, Guangzhou 510642, China; 2 College of Forestry, Northwest A&F University, Yangling 712100, China; 3 Boone Pickens School of Geology, Oklahoma State University, Stillwater, OK 74078, USA

**Keywords:** arbuscular mycorrhizal fungi, *Casuarina glauca*, *CLC*, *NHX*, salt stress

## Abstract

*Casuarina glauca* is an important coastal protection forest species, which is exposed to high salt stress all year round. Arbuscular mycorrhizal fungi (AMF) can promote the growth and salt tolerance of *C*. *glauca* under salt stress. However, the effects of AMF on the distribution of Na^+^ and Cl^−^ and the expression of related genes in *C*. *glauca* under salt stress need to be further explored. This study explored the effects of *Rhizophagus irregularis* on plant biomass, the distribution of Na^+^ and Cl^−^, and the expression of related genes in *C*. *glauca* under NaCl stress through pot simulation experiments. The results revealed that the mechanisms of Na^+^ and Cl^−^ transport of *C. glauca* under NaCl stress were different. *C. glauca* took a salt accumulation approach to Na^+^, transferring Na^+^ from roots to shoots. Salt accumulation of Na^+^ promoted by AMF was associated with *CgNHX7*. The transport mechanism of *C. glauca* to Cl^−^ might involve salt exclusion rather than salt accumulation, and Cl^−^ was no longer transferred to shoots in large quantities but started to accumulate in roots. However, AMF alleviated Na^+^ and Cl^−^ stress by similar mechanisms. AMF could promote salt dilution of *C. glauca* by increasing biomass and the content of K^+^, compartmentalizing Na^+^ and Cl^−^ in vacuoles. These processes were associated with the expression of *CgNHX1*, *CgNHX2-1*, *CgCLCD*, *CgCLCF*, and *CgCLCG.* Our study will provide a theoretical basis for the application of AMF to improve salt tolerance in plants.

## 1. Introduction

Salt stress is one of the most severe abiotic stresses that the world is facing [1]. Approximately 800 million hectares of land and approximately 20% of irrigated land in the world are under some degree of salt stress [2,3]. The ionic toxicity of salt stress can induce osmotic stress in plants, leading to imbalanced nutrition and even plant death [4]. NaCl stress is one of the most extensive and damaging salt stresses [5,6]. However, halophytes can grow under high salt stress [7,8]. There are three methods related to their salt tolerance under salt stress. Salt exclusion is common in pseudohalophytes. Their roots rarely absorb salt or mainly accumulate the absorbed salt in the roots, so the ion concentration in the leaves is significantly lower than that in the roots within a certain concentration range [9,10]. Salt excretion is common in recretohalophytes. They have salt glands, which can actively secrete the absorbed salt to the surface of the stems and leaves for the rain to wash away [11]. Salt dilution is common in euhalophytes. They reduce salt concentration by rapidly growing cells to absorb water, increasing osmotic adjustment substances, and compartmentalizing salt in vacuoles [12,13]. It would be interesting to explore the mechanism of salt tolerance in these plants and then apply the insights gained to improve the yields of crops under salt stress.

Arbuscular mycorrhizal fungi (AMF) form symbiotic relationships with more than 90% of terrestrial plants and 80 to 90% of vascular plants [14]. AMF symbionts may be one of the most extensive beneficial interactions between plants and microorganisms [15], which occur naturally, even in salty environments where they promote the growth of mycorrhizal plants [16,17]. Mycorrhization can enhance the absorption of water and nutrients [18,19], the accumulation of osmotic adjustment substances, and the activity of antioxidant enzymes in plants [20]. Furthermore, mycorrhization can reduce the concentrations of salt ions in plants under salt stress [21,22]. Most studies have focused on investigating plant growth promotion upon mycorrhization under salt stress [23,24].

*Casuarina glauca* is native to Australia, Southeast Asia, and the Pacific islands, and was introduced to the southeast coastal areas of China in the 1950s [25]. Due to its tolerance to drought, infertile soil, salt, and sandstorms, *C. glauca* has become one of the main forest tree species in coastal zones by playing an irreplaceable role in windbreak and sand fixation. Hence, it helps to improve the ecological environment by providing materials [26,27]. As a coastal shelterbelt species, *C. glauca* is mainly exposed to Na^+^ and Cl^−^ toxicity [28,29]. Djighaly et al. [30] found that *C. glauca* was more tolerant to NaCl stress than *C. equisetifolia*, and inoculation with AMF could improve the uptake of nutrients, such as N and P, and water uptake by *C. glauca* under NaCl stress. The results of Djighaly et al. [30] indicated that inoculation with AMF increased the growth of *C. glauca* under NaCl stress, with a positive effect on the diversity of herbaceous vegetation around it, which could be used to reduce the NaCl concentration in the soil. Our previous study also found that AMF inoculation under NaCl stress plays an important role in promoting growth, regulating ion balance, and changing the activity of antioxidant enzymes in *C. glauca* [31]. In addition, NHXs (Na^+^/H^+^ exchangers) and CLCs (the chloride channels), related to Na^+^ and Cl^−^ transport, are also related to *C. glauca*’s NaCl tolerance [32,33].

NHXs play important roles in regulating ion homeostasis and cell growth [34]. They exchange H^+^ for Na^+^ or K^+^, thereby regulating monovalent cation homeostasis [35]. *Arabidopsis thaliana* contains eight NHX isoforms [36]. NHX1 to NHX4, located in the tonoplast, are associated with growth and vacuolar K^+^ homeostasis [37]. NHX5 and NHX6, which are located in the Golgi and trans-Golgi networks (TGNs), are thought to facilitate Na^+^ (K^+^)/H^+^ exchange [38] and growth [39]. NHX7 (salt overly sensitive 1, SOS1) and NHX8, which are located in the plasma membrane, are involved in Na^+^ (Li^+^)/H^+^ exchange, respectively transporting Na^+^ and Li^+^ out of the cell [40].

The CLC protein family, with its dual functions of NO^3−^ and Cl^−^ transport, plays an important role in salt tolerance and nutrient absorption [33]. *A. thaliana* contains seven CLC isoforms [41]. CLCA and CLCB, located in the tonoplast, are associated with NO^3−^/H^+^ exchange [42]. As a Cl^−^ channel located in the tonoplast, CLCC plays an important role in regulating stomatal movement, anion homeostasis, and salt tolerance [43]. CLCD and CLCF are localized in Golgi membranes [41]. CLCD is associated with root growth as a Cl^−^ channel [44]. CLCE, which targets the thylakoid membranes in chloroplasts, is related to photosynthesis [45]. Some authors have proposed that CLCF has the same function as CLCE [33], whereas others consider CLCF to be more similar to CLCD [46]. CLCG, located in the tonoplast, is considered to have the same function as CLCC [47].

Most studies on AMF and salt stress have focused on the distribution of Na^+^ and the expression of related genes. The effects of AMF on the distribution of both Na^+^ and Cl^−^, and the expression of related genes need to be further explored. This study has three aims:

1. To explore the influence of mycorrhization on the growth and distribution of Na^+^ and Cl^−.^
2. To explore the influence of mycorrhization on the gene expression of the *CgNHX* and *CgCLC* families.
3. To explore whether these genes are related to Na^+^ and Cl^−^ distribution.

## 2. Results

### 2.1. Effect of Rhizophagus irregularis on Mycorrhizal Colonization and Plant Biomass under NaCl Stress

Figure S1 shows the colonization of arbuscule, hypha, vesicle, and spore in mycorrhizal *C. glauca* under NaCl stress. With a change in NaCl concentration from 0 to 600 mM, arbuscular colonization decreased from 39% to 14%, vesicle and spore colonization increased from 34% to 57%, and hyphal colonization exhibited no significant change.

Under NaCl stress, the biomass of *C. glauca* differed between plants with *R. irregularis* inoculation and those without. Figure S2 shows that NaCl stress inhibited the growth of *C. glauca* and *R. irregularis* inoculation alleviated this inhibition (Figure S2a). NaCl stress reduced the fresh weight, ground diameter, and height of *C. glauca* (Figure S2b–d). The inoculation with *R. irregularis* could promote the growth of *C. glauca* regardless of the presence of NaCl stress, specifically by increasing the fresh weight, plant height, and ground diameter of *C. glauca* (Figure S2b–d). These results show that *R. irregularis* inoculation can promote the growth and salt tolerance of *C. glauca* under NaCl stress.

### 2.2. Effect of R. irregularis on Na^+^, K^+^, and Cl^−^ Status under NaCl Stress

**Figure 1:**
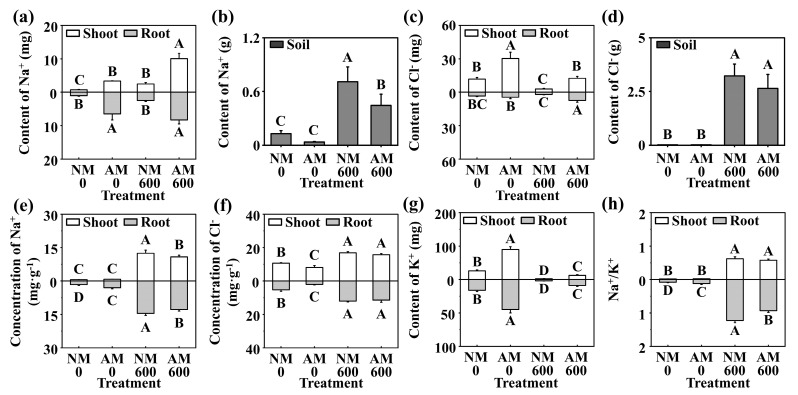
Effect of *R. irregularis* on Na^+^, K^+^, and Cl^−^ status under NaCl stress. (**a**) The content of Na^+^ in *C. glauca*. (**b**) The content of Na^+^ in soil. (**c**): The content of Cl^−^ in *C. glauca*. (**d**) The content of Cl^−^ in soil. (**e**) The concentration of Na^+^ in *C. glauca*. (**f**) The concentration of Cl^−^ in *C. glauca*. (**g**) The content of K^+^ in *C. glauca*. (**h**) The Na^+^/K^+^ of *C. glauca*. NM, nonmycorrhizal; AM, inoculated with *R. irregularis*; 0, no NaCl stress; 600, 600 mM NaCl stress. The data are the means ± standard errors (*n* = 3). Different capital letters indicate significant differences between means at the *p* < 0.05 level by Tukey’s test. Data of roots from Wang et al. [31] in the same project.

The effects of *R. irregularis* inoculation on the Na^+^ and Cl^−^ contents under NaCl stress were consistent, and both were increased. Therefore, the contents in the soil were reduced (Figure 1a–d). The Na^+^ and Cl^−^ concentrations of *C. glauca* were increased by NaCl stress. Instead, under NaCl stress, inoculation with *R. irregularis* decreased the Na^+^ and Cl^−^ concentrations of shoots and roots (Figure 1e,f). The K^+^ content of *C. glauca* was reduced by NaCl stress, but inoculation with *R. irregularis* increased the K^+^ content of shoots and roots under NaCl stress (Figure 1g). The Na^+^/K^+^ of *C. glauca* was increased by NaCl stress. Nevertheless, under NaCl stress, *R. irregularis* inoculation decreased the Na^+^/K^+^ of shoots and roots by 8% and 24%, respectively (Figure 1h).

**Figure 2:**
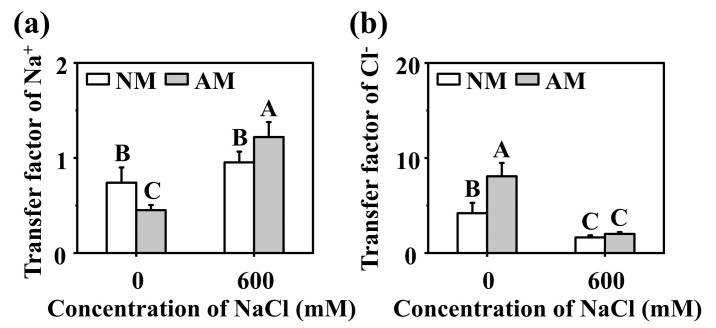
The transfer factor (TF) of Na^+^ and Cl^−^ in *C. glauca* under different treatments. (**a**) The TF of Na^+^ in *C. glauca*. (**b**) The TF of Cl^−^ in *C. glauca*. NM, nonmycorrhizal; AM, inoculated with *R. irregularis*; 0, no NaCl stress; 600, 600 mM NaCl stress. The data are the means ± standard errors (*n* = 3). Different capital letters indicate significant differences between means at the *p* < 0.05 level by Tukey’s test.

The effects of NaCl stress on the TF of Na^+^ and Cl^−^ were different, increasing the TF of Na^+^ but decreasing that of Cl^−^. The effects of *R. irregularis* inoculation on the Na^+^ and Cl^−^ TF under NaCl stress were consistent, and both were increased (Figure 2).

### 2.3. Effect of R. irregularis on Gs and Tr under NaCl Stress

**Figure 3:**
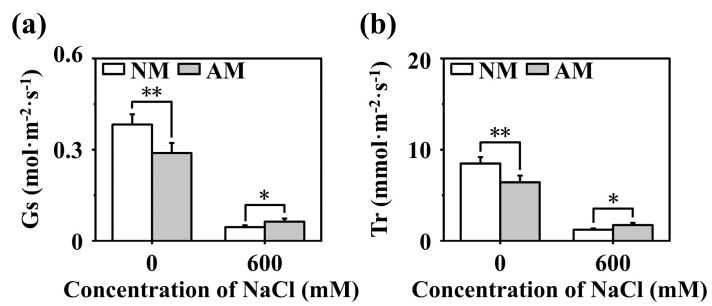
The stomatal conductance (Gs) and transpiration rate (Tr) in leaves of *C. glauca* in different treatments. (**a**) The Gs in leaves of *C. glauca* in different treatments. (**b**) The Tr in leaves of *C. glauca* in different treatments. NM, nonmycorrhizal; AM, inoculated with *R. irregularis*; 0, no NaCl stress; 600, 600 mM NaCl stress. The data are the means ± standard errors (*n* = 3). * indicates significant differences between means at the *p* < 0.05 level by one-way ANOVA, and ** indicates significant differences between means at the *p* < 0.01 level by one-way ANOVA.

To explore whether the increase of TF of Na^+^ and Cl^−^ caused by *R. irregularis* inoculation is related to stomatal conductance (Gs) and transpiration rate (Tr), we investigated the effect of *R. irregularis* on Gs and Tr under NaCl Stress. The results showed that NaCl stress decreased Gs and Tr. Under NaCl stress, compared with no inoculation, inoculation with *R. irregularis* increased Gs and Tr by 31% and 26%, respectively. (Figure 3).

### 2.4. Effect of R. irregularis on Expression of CgNHXs and CgCLCs under NaCl Stress

To explore whether the effect of *R. irregularis* inoculation on Na^+^, K^+^, and Cl^−^ status is related to Na^+^ and Cl^−^ transport-related genes, we determined the expression of *CgNHXs* and *CgCLCs*.

**Figure 4:**
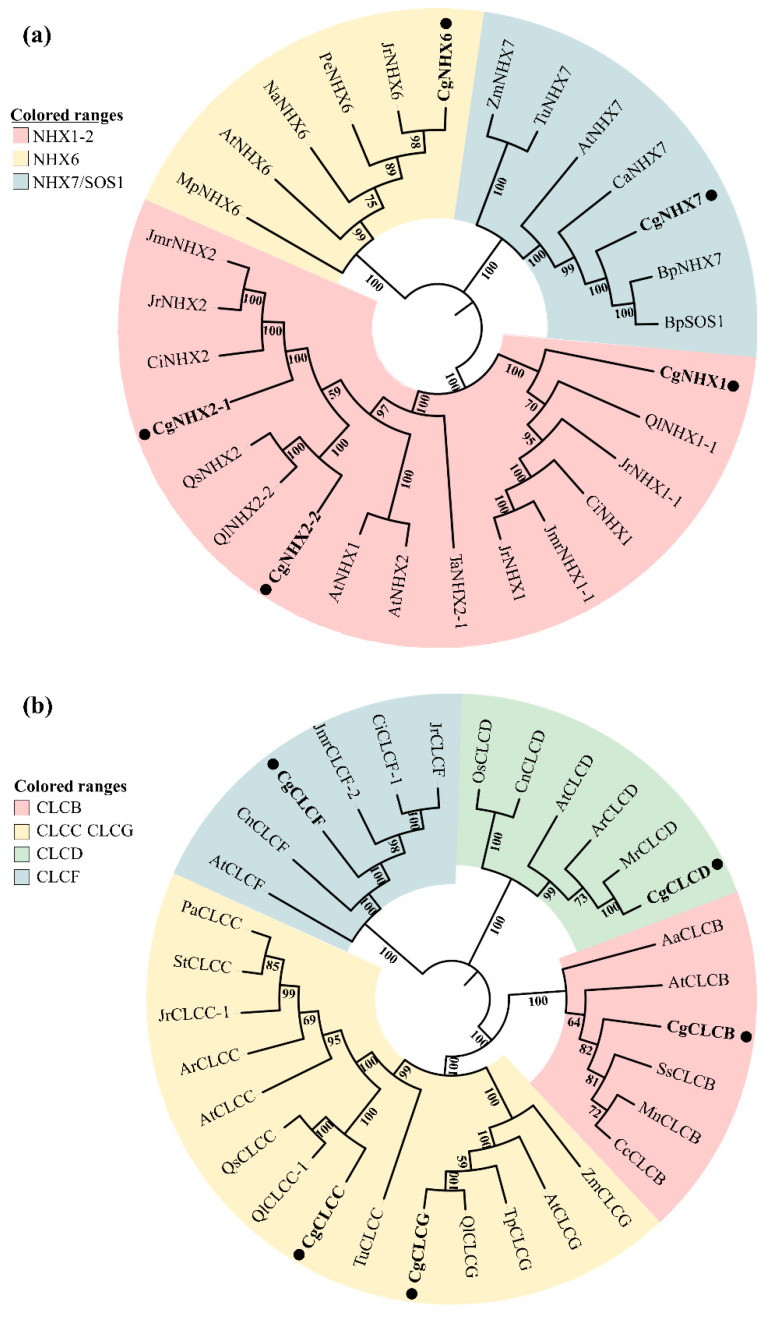
Phylogenetic tree of NHX and CLC protein translated from cloned genes. (**a**) Phylogenetic tree of NHX protein. (**b**) Phylogenetic tree of CLC protein. The genes marked by black dots are the target genes of the study. The NCBI number of the cloned gene is as follows: ON246321 (*CgNHX1*), ON246322 (*CgNHX2-1*), ON246323 (*CgNHX2-2*), ON246324 (*CgNHX6*), ON246325 (*CgNHX7*), ON206670 (*CgCLCB*), ON246326 (*CgCLCC*), ON246328 (*CgCLCD*), ON246329 (*CgCLCF*), ON246330 (*CgCLCG*). The sequences of *Actinidia rufa*, *Arabidopsis thaliana*, *Artemisia annua*, *Betula platyphylla*, *Carya illinoinensis*, *Citrus clementina*, *Cocos nucifera*, *Cucurbita argyrosperma*, *Juglans microcarpa × Juglans regia*, *Juglans regia*, *Morella rubra*, *Morus notabilis*, *Mucuna pruriens*, *Nicotiana attenuate*, *Oryza sativa*, *Populus euphratica*, *Prosopis alba*, *Quercus lobata*, *Quercus suber*, *Senna tora*, *Spatholobus suberectus*, *Trifolium pratense*, *Triticum aestivum*, *Triticum Urartu*, *Zea mays* used for phylogenetic tree building were all from NCBI.

A total of five full-length *CgNHX* genes and five full-length *CgCLC* genes were obtained by cloning. These full-length translated protein sequences were compared with NHX and CLC proteins of known species such as *Arabidopsis thaliana* and *Zea mays,* and a phylogenetic tree was constructed to initially determine their potential functions. As shown in Figure 4a, the five NHX proteins were divided into three branches. Among them, CgNHX1, CgNHX2-1, and CgNHX2-2 were classified into the same branch with a closer affinity to AtNHX1 and AtNHX2. CgNHX6 was classified into the second branch with closer affinity to AtNHX6. CgNHX7 was classified into the third branch with a closer affinity to AtNHX7. As shown in Figure 4b, the five CLC proteins were divided into four branches. Among them, CgCLCB was divided into the first branch, which is more closely related to AtCLCB. CgCLCC and CgCLCG were divided into the second branch, which is more closely related to AtCLCC and AtCLCG. CgCLCD was divided into the third branch, which is more closely related to AtCLCD. CgCLCF was divided into the fourth branch, which is more closely related to AtCLCF.

**Figure 5:**
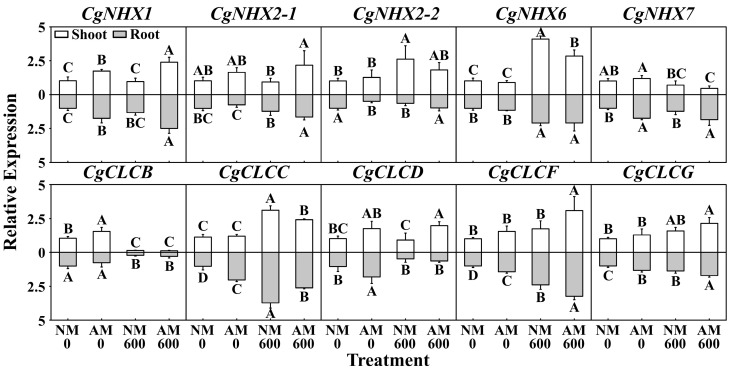
Effects of NaCl stress and *R. irregularis* inoculation on the expression of *CgNHXs* and *CgCLCs* in shoots and roots. NM, nonmycorrhizal; AM, inoculated with *R. irregularis*; 0, no NaCl stress; 600, 600 mM NaCl stress. Different capital letters indicate significant differences between means at the *p* < 0.05 level by Tukey’s test.

The patterns of *CgNHX* expression differed between shoots and roots. In shoots, the expression of *CgNHX1* and *CgNHX2-1* was upregulated by *R. irregularis* inoculation. The expression of *CgNHX2-2* and *CgNHX6* was upregulated by NaCl stress but downregulated by *R. irregularis* inoculation under NaCl stress. In addition, the expression of *CgNHX7* was downregulated by NaCl stress. In roots, the expression of *CgNHX1*, *CgNHX2-1*, and *CgNHX2-2* was upregulated by *R. irregularis* inoculation under NaCl stress. The expression of *CgNHX6* was upregulated by NaCl stress. Under NaCl stress, the expression of *CgNHX7* was upregulated by *R. irregularis* inoculation (Figure 5).

The patterns of *CgCLC* expression differed between shoots and roots. In shoots, the expression of *CgCLCB* was downregulated by NaCl stress. The expression of *CgCLCC* was upregulated by NaCl stress but downregulated by *R. irregularis* inoculation under NaCl stress. In addition, the expression of *CgCLCD*, *CgCLCF*, and *CgCLCG* was upregulated by *R. irregularis* inoculation under NaCl stress. In roots, the expression of *CgCLCB* and *CgCLCD* was downregulated by NaCl stress, whereas the expression of *CgCLCC*, *CgCLCF*, and *CgCLCG* was upregulated by NaCl stress. *R. irregularis* inoculation downregulated the expression of *CgCLCC* but upregulated *CgCLCF* and *CgCLCG* under NaCl stress (Figure 5).

### 2.5. Correlation Analysis of Gene Expression and Physiological Indicators of C. glauca under NaCl Stress

To explore the correlation between the effect of *R. irregularis* inoculation on physiological indicators and the Na^+^, Cl^−^ transport-related genes under NaCl stress, we did a correlation analysis between them under salt stress.

**Figure 6:**
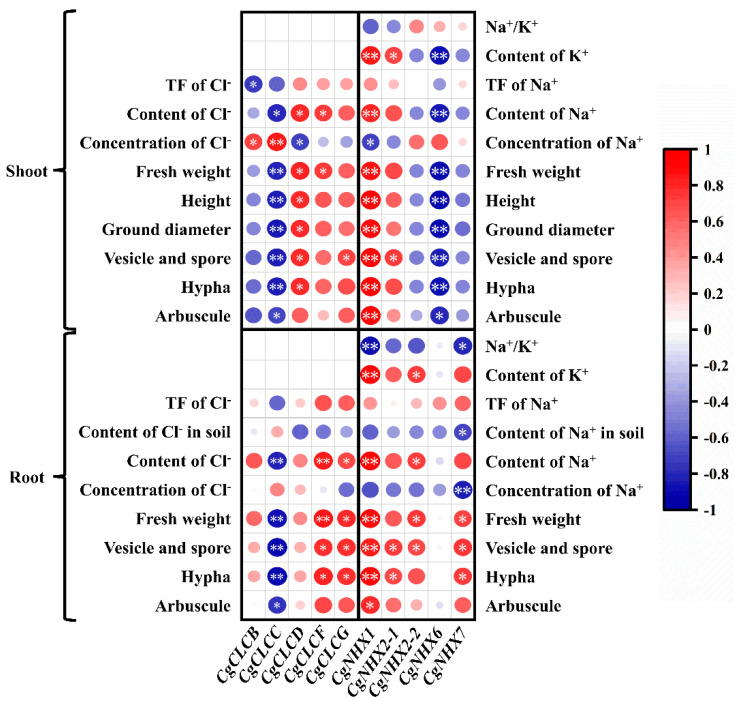
Correlation coefficients of gene expression and physiological indices of *C. glauca* under NaCl stress. Each circle indicates Pearson’s correlation coefficient of a pair of parameters. * indicates a significant difference between the correlation coefficients at the *p* < 0.05 level, ** indicates a significant difference between the correlation coefficients at the *p* < 0.01 level.

Opposite to *CgNHX6*, the correlation analysis results of the expression of *CgNHX1* in shoots and roots and *CgNHX2-2* in roots were all positively correlated with mycorrhizal colonization, biomass, and the content of Na^+^, K^+^, except that the expression of *CgNHX1* in shoots was negatively correlated with the concentration of Na^+^ and that in roots was negatively correlated with Na^+^/K^+^. The expression of *CgNHX2-1* in shoots was positively correlated with mycorrhizal colonization and the content of K^+^, and that in roots was only positively correlated with mycorrhizal colonization. The expression of *CgNHX7* in roots was positively correlated with mycorrhizal colonization and biomass but was negatively correlated with the concentration of Na^+^, the content of Na^+^ in soil, and Na^+^/K^+^ (Figure 6).

Correlation analysis showed that the expression of *CgCLCB* in shoots was positively correlated with the concentration of Cl^−^ and negatively correlated with the TF of Cl^−^. However, the expression of *CgCLCC* in shoots was positively correlated with the concentration of Cl^−^ and negatively correlated with other indicators, which was the opposite of the expression of *CgCLCD* in shoots. The expression of *CgCLCC* in roots was negatively correlated with mycorrhizal colonization, fresh weight, and Cl^−^ content. Both the expression of *CgCLCF* in shoots and roots were positively correlated with fresh weight and the content of Cl^−^. In addition, the expression of *CgCLCF* in roots was also positively correlated with mycorrhizal colonization. The correlation analysis results of *CgCLCF* in roots were consistent with those of *CgCLCG* in roots (Figure 6).

## 3. Discussion

### 3.1. Effects of R. irregularis Inoculation and NaCl Stress on Colonization and Biomass

Salt stress inhibited plant growth and AMF growth in the same way [48,49]. Soil salinity had a negative correlation with the intensity of mycorrhizal colonization on the one hand, and a positive correlation with spore density on the other hand. We believed that these results reflect the assistance of *R. irregularis* to plants resisting NaCl stress [22]. Sporulation was considered as a resistance behavior exhibited by mycorrhization against high salt concentrations [21].

The most significant and common effect of salt on plants is growth inhibition, since plants need to absorb water and ions to maintain osmotic pressure, synthesize osmotic adjustment substances, and compartmentalize harmful ions to survive. These processes require energy, resulting in less energy required for growth and lower biomass [50,51]. AMF could increase energy by enhancing the ability of plants to absorb water, synthesize osmotic adjustment substances, and compartmentalize harmful ions, thereby promoting plant growth [52,53]. Additionally, we regarded the significant increase of *C. glauca* biomass caused by *R. irregularis* inoculation as an important indicator of the ability of plants to tolerate salinity stress [53,54].

### 3.2. Effects of R. irregularis Inoculation and NaCl Stress on Na^+^, K^+^, and Cl^−^ Uptake

Many studies have focused only on the tolerance mechanism of Na^+^ under salt stress, and have assumed that Na^+^ can represent NaCl. However, our results showed that the change trends of Na^+^ and Cl^−^ TF were different under NaCl stress, reflecting the different salt tolerance mechanisms of the two. For Na^+^, the Na^+^ TF increased under NaCl stress, which meant that the tolerance mechanism of Na^+^ in *C. glauca* might be salt accumulation, and hence a large amount of Na^+^ accumulated in the shoots [55,56]. The shoots were then protected by salt dilution, increasing tissue water content, or compartmentalizing Na^+^ to the vacuole [13,57]. However, for Cl^−^, the TF of Cl^−^ was significantly decreased under NaCl stress. At the same time, the Cl^−^ content in shoots was still greater than in roots. This meant that under the stress of 600 mM NaCl, the tolerance mechanism of *C. glauca* to Cl^−^ might involve salt exclusion rather than salt accumulation [58]. Perhaps Cl^−^ is more toxic to shoots than Na^+^ for *C. glauca*, or the concentration of Cl^−^ in shoots reaches the tolerance threshold, but Na^+^ concentration does not [59,60].

Although the mechanisms of *C. glauca* tolerance to Na^+^ and Cl^−^ were different, the mitigation mechanisms of *R. irregularis* inoculation to their stress were similar. The upward transport of Na^+^ and Cl^−^ improved by AMF was caused by transpiration flow in plants under salt stress [61]. The transpiration flow was caused by increases in Gs and Tr, which were attributed to *R. irregularis* inoculation. AMF could promote salt dilution of halophytes by increasing plant biomass and the content of osmotic adjustment substances, compartmentalizing Na^+^ and Cl^−^ [62,63]. Due to the increase in biomass caused by *R. irregularis* inoculation, mycorrhizal *C. glauca* could simultaneously maintain high contents and low concentrations of Na^+^ and Cl^−^. This is a common feature of AMF-associated plants under salt stress [26,64]. Compared with nonmycorrhizal *C. glauca*, mycorrhizal *C. glauca* absorbed larger amounts of Na^+^ and Cl^−^ from the soil but maintained lower concentrations of Na^+^ and Cl^−^ to protect themselves from excessive damage. Meanwhile, mycorrhizal plants had lower Na^+^/K^+^ to regulate the dynamic equilibrium of ions and improve salt tolerance [65,66]. Therefore, even if increased Gs and Tr promotes the upward transport of Na^+^ and Cl^−^, the mycorrhizal *C. glauca* does not suffer severe ion damage.

### 3.3. Effects of R. irregularis Inoculation and NaCl Stress on Gene Expression

Roots are the first plant part to be stressed during salt stress. The AM symbiosis increased the saline stress tolerance of *Robinia pseudoacacia* through the upregulation of *SOS1/NHX7* in roots, which enhanced the exclusion of Na^+^ from root cells [67]. This indicated that *R. irregularis* inoculation promoted Na^+^ efflux from roots and prevented Na^+^ poisoning. Meanwhile, *R. irregularis* inoculation decreased the Na^+^ content in the soil and increased TF of Na^+^, implying that possibly that most of the Na^+^ efflux in roots upon induction of *CgNHX7* by *R. irregularis* inoculation was transferred to the shoots rather than the soil. Meanwhile, under NaCl stress, the expression of *CgNHX7* decreased in shoots but increased in roots. This is consistent with our previous notion (see above) that *R. irregularis* inoculation might promote the salt accumulation of Na^+^ in shoots. We mentioned earlier that AMF could promote salt dilution of halophytes by increasing plant biomass and the content of osmotic adjustment substances, compartmentalizing Na^+^.

So, are these processes co-regulated with the expression of *CgNHXs*?, since vacuolar *NHXs*, *NHX1* and *NHX2* could promote the transport of Na^+^ to vacuoles [53]. Hence, the upregulation of *CgNHX1* and *CgNHX2-1* caused by *R. irregularis* inoculation might have alleviated the inhibitory effects of Na^+^ by transporting it to vacuoles. Furthermore, similar to our correlation analysis results, *NHX1* and *NHX2* had the same functions in growth-promoting and the stabilization of K^+^ in vacuoles [35,68]. Therefore, the upregulation of *CgNHX1* and *CgNHX2-1* by *R. irregularis* inoculation might not only increase the biomass of mycorrhizal *C. glauca* to reduce Na^+^ toxicity by bio-dilution, but also stabilize the cellular environment by increasing the K^+^ content in shoots and roots.

Which *CgCLCs* can mycorrhizal *C. glauca* use for salt dilution? The expression of *CgCLCC* in *C. glauca* under NaCl stress was higher than in those without NaCl stress treatment, and plants with high Cl^−^ toxicity protect themselves by upregulating the expression of *CLCC* to transport Cl^−^ into vacuoles [69]. Another *CLC* gene, *AtCLCG*, is involved in salt tolerance by altering Cl^−^ homeostasis in mesophyll cells and Cl^−^ compartmentalization into the vacuole. This gene is co-expressed with *AtCLCC* in guard cells during stomatal regulation [47]. Although *R. irregularis* inoculation downregulated *CgCLCC* under salt stress, it upregulated *CgCLCG,* which might promote the transfer of Cl^−^ into mesophyll cell vacuoles, thereby improving tolerance of mycorrhizal *C. glauca* against NaCl stress, and promoting its growth. In addition, *CLCD*, an anion transporter involved in compartments and trafficking, might be related to growth and Cl^−^ transfer into the trans-Golgi network [33]. The function of another *CLC* gene, *CgCLCF,* might be similar to that of *CgCLCD*, which was correlated with Cl^−^ transport and growth. The increase in biomass and Cl^−^ compartmentalization upon *R. irregularis* inoculation might have been related to *CgCLCD* and *CgCLCF* induction.

## 4. Materials and Methods

### 4.1. Experimental Design and Materials

This study was a two-factor experiment: NaCl stress (0 mM NaCl and 600 mM NaCl) and AMF inoculation (no inoculation with AMF and inoculation with *Rhizophagus irregularis*). There were four treatments, and three biological replicates for each treatment. Each replicate contained 12 pots, for a total of 144 pots.

*C. glauca* seeds were sterilized and germinated into seedlings, and the best-growing plant was selected from the seedlings to prepare a batch of tissue culture seedlings, which were used as experimental materials [31]. *R. irregularis* was multiplied by *Lycopersicon esculentum* Mill and then triturated, and 40 g of inoculum was added to each pot for *R. irregularis* inoculation treatment, and 40 g of sterilized inoculum was added to each pot for no AMF treatment. Seedlings were grown in greenhouse at temperatures ranging from 25–35 °C and relative humidity ranging from 50–70%.

### 4.2. Plant Harvest

The stomatal conductance (Gs) and transpiration rate (Tr) were measured with a Li-6400 portable open flow gas-exchange system (Li-Cor Inc., Lincoln, NE, USA) between 8:30 to 11:30 a.m. The method and parameter settings were those of Sheng et al. [70].

After the shoots were weighed for total fresh weight, they were divided into three parts. The first part was used to calculate the water content, the second was used for elemental concentration determination, and the third was used to extract RNA. After weighing the total fresh weight of roots, the grouping and uses were the same as those of shoots, except that a part was separated to measure AMF colonization.

### 4.3. Mycorrhizal Colonization

Refer to Phillips and Hayman [71] and Giovannetti and Mosse [72] for the determination method of AMF colonization.

### 4.4. Plant Biomass and Water Content

The total fresh weight of shoots and roots was weighed at harvest time. The plant height was measured with a tape measure, and the ground diameter was measured with a digital caliper (Hengliang, Shanghai, China). The part of the sample used to measure the water content was weighed fresh and then dried at 65 °C after fixation at 105 °C to measure the dry weight. The water content was calculated using the dry and fresh weights. Total dry weight was calculated using water content and total fresh weight.

### 4.5. Na^+^, K^+^, and Cl^−^ Status

After being ground, the part of the sample used to measure the Na^+^ and K^+^ concentration was extracted with HNO_3_ and a microwave digestion instrument (Milestone ETHOS, Milan, Italy). The elements were measured by atomic absorption spectrophotometer (PerkinElmer, Waltham, MA, USA). The measurement method of Cl^−^ concentration was that of Matsushita and Matoh [73] and Zhan et al. [74]. The contents of the elements were calculated by the concentration and total dry weight. The extraction method of elements in the soil was that of Bao [75]. The method for measuring elements in soil was the same as above. The transfer factor (TF) of Na^+^ and Cl^−^ was obtained by dividing the shoot content by the root content.

### 4.6. RNA Extraction, Complementary DNA (cDNA) Synthesis, and Determination of Gene Expression via Quantitative Real-Time PCR (qRT–PCR)

RNA extraction used the E.Z.N.A Plant RNA Kit R6827-01 (Omega Bio-Tek, Norcross, GA, USA). RNA quality check used 1% agarose gel electrophoresis and a NanoDrop 2000 instrument (Thermo Scientific, Pittsburgh, PA, USA). Reverse transcription of RNA was done using the TIANScript RT Kit (TIANGEN Bio, Beijing, China) to obtain cDNA. The sequences of *CgNHXs* and *CgCLCs* were obtained from the assembly of the *C. glauca* transcriptome sequenced by Illumina HiSeq technology (PRJNA690646). The full-length gene was cloned using the SMARTer^®^ RACE 5′/3′ Kit (Takara Bio, Beijing, China). The primers used for qRT–PCR were designed with the NCBI Primer-BLAST tool (Table S1). qRT–PCR was conducted using a CF96X real-time PCR system (Bio-Rad, Hercules, CA, USA). The specific reaction system and steps were consistent with previous research [31]. There were three biological replicates for each treatment, and two technical replicates for each biological replicate. The relative quantity of transcripts was determined using the $2^{-\Delta \Delta C_{T}}$ method [76].

### 4.7. Statistical Analyses

SPSS 26 statistical software (SPSS Inc., Chicago, IL, United States) was used for statistical analyses. All data were subjected to one-way ANOVA and post hoc comparisons (Tukey’s test, *p* < 0.05, *n* = 3). Figures were constructed with Origin 2020 (Origin Lab, Northampton, MA, USA.). The phylogenetic tree was plotted using ITOL (https://itol.embl.de/itol.cgi, accessed on 19 January 2023).

## 5. Conclusions

Under NaCl stress, the tolerance mechanisms of *C. glauca* to Na^+^ and Cl^−^ were different. For Na^+^, salt accumulation was adopted, and Na^+^ absorbed from the soil was transferred from roots to shoots. This process was promoted by *R. irregularis* inoculation and was related to Gs, Tr, and *CgNHX7* expression. The Cl^−^ tolerance mechanism involved salt exclusion, and Cl^−^ absorbed from soil was no longer transferred to shoots in large quantities but started accumulating in roots. However, the mechanism of *R. irregularis* inoculation alleviating Na^+^ and Cl^−^ stress was similar, promoting growth, increasing osmotic adjustment substances, and ionic compartmentalization. Therefore, mycorrhizal *C. glauca* could not only absorb large amounts of Na^+^ and Cl^−^ from the soil to remediate it but also mitigate Na^+^ and Cl^−^ stress in plants. The process of *R. irregularis* inoculation alleviating Na^+^ and Cl^−^ stress was correlated with the expression of *CgNHX1*, *CgNHX2-1*, and *CgCLCD*, *CgCLCF*, *CgCLCG*. Future studies may focus on the specific effects of *R. irregularis* inoculation on these genes and the detailed Na^+^ and Cl^−^ transfer pathways in *C. glauca* under NaCl exposure.

## Data Availability

The data that support the findings of this study are available on request from the corresponding author.

## Supplementary Materials

The following supporting information can be downloaded at: https://www.mdpi.com/article/10.3390/ijms24043680/s1.
