# Supplementary material for: Effects of Arbuscular Mycorrhizal Fungus on Sodium and Chloride Ion Channels of Casuarina glauca under Salt Stress
Source: Int J Mol Sci. 2023 Feb 12;24(4):3680. doi: 10.3390/ijms24043680 (PMC9966195; doi:10.3390/ijms24043680)
Supplement: Supplementary file 1 [file ijms-24-03680-s001.zip › ijms-2150590-supplementary.pdf]

International Journal of
Molecular Sciences

MDPI

Supplementary Material

Table S1. Primers used for quantitative real-time PCR (qRT-PCR).

Gene Name	Forward Primer	Reverse Primer	Tm (°C)
CgNHX1	TTCTTCCTTTATCTGCTTCCTCCAA	GCTCCAAACAAAAAGATAGTCGTGA	50.8
CgNHX2-1	GTGGTTTCCATGAACCTCTTTGTAG	TCACTAAACACTAAAAGATGCGAGC	53.5
CgNHX2-2	TTGGTTTGTGTACCGGAGTAGTTAT	CGGCGGAAGAAGATAAATGAAGAAA	48.0
CgNHX6	CAATGGCTTTTGCCCTTGCT	GCTTCCAGCATAGTGCCTGT	61.0
CgNHX7	ATTTCTCCGTGCCCATCGAA	TCTTGTCTTCACAACCCGCA	61.0
CgCLCB	TGGGAAGCCTTTACAACTACTTTCT	TGCATTTAACGAGAAAAGGAAGACC	50.8
CgCLCC-1	ATTCCTGTCATCCTTGCGGG	ATGATGCAGCTCCGAGAAGG	55.7
CgCLCD	ATTGCTGGGAGCTGCTTCTT	CCCTCAGACGTGCTTGTTCT	55.7
CgCLCF	ATGACAAACCGGCCAAAGGA	GCGGAACCTTCACACAATCT	55.7
CgCLCG	TTCGGTCTCTAATTTGGTTCTGACT	TAGTGATGATGAAACCGCAGAAATG	50.8
CgEF1-α	CTTAACAAATCCAGCATCACC	CAGTCCTTGATTGCCACAC	59.0
CgTUB	TGTCAGTGGAGCAAACCCAA	CTATGACATTTGCTTCCGAAC	63.3

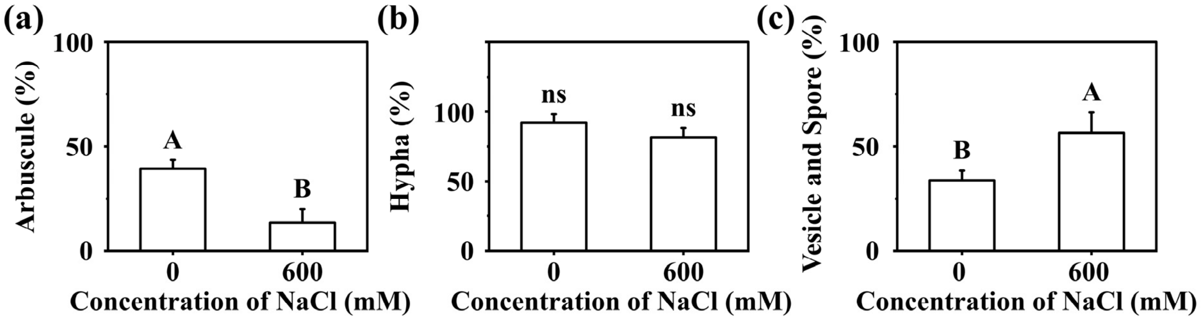

Figure S1. *R. irregularis* colonization rates of *C. glauca* under different treatments. (a): Colonization rates of arbuscule. (b): Colonization rates of hypha. (c): Colonization rates of vesicle and spore. 0, no NaCl stress; 600, 600 mM NaCl stress. The data are the means ± standard errors (n = 3). Different capital letters indicate significant differences between means at the $P < 0.05$ level by Tukey's test, and "ns" indicates no significant difference. Data in the graph from Wang et al. (2022) in the same project.

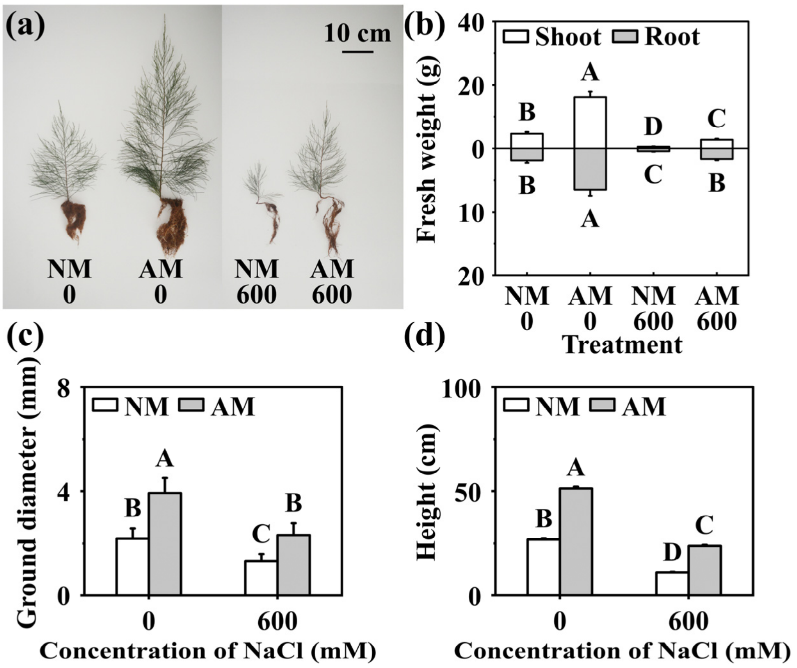

Figure S2. Biomass of *C. glauca* under different treatments. (a): Growth phenotype of *C. glauca* under different treatments. (b) Fresh weight of *C. glauca*. (c): Ground diameter of *C. glauca*. (d) Height of *C. glauca*. NM, nonmycorrhizal; AM, inoculated with *R. irregularis;* 0, no NaCl stress; 600, 600 mM NaCl stress. The data are the means ± standard error (n = 3). Different capital letters indicate significant differences between means at the $P < 0.05$ level by Tukey's test. Data in the graph from Wang et al. (2022) in the same project.
